# Simple automation of SEM‐EDS spectral maps analysis with Python and the edxia framework

**DOI:** 10.1111/jmi.13099

**Published:** 2022-03-24

**Authors:** Fabien Georget, William Wilson, Karen L. Scrivener

**Affiliations:** ^1^ Laboratory of Construction Materials EPFL Lausanne Switzerland; ^2^ Institute of Building Materials RWTH Aachen Aachen Germany; ^3^ Université de Sherbrooke Sherbrooke Quebec Canada

**Keywords:** automation, cement paste, edxia, image analysis, SEM‐EDX

## Abstract

In a recent article, we described the *edxia* framework, a user‐friendly framework to analyse the microstructure of cementitious materials using SEM‐EDS hypermaps. The manual approach presented was shown to be efficient to answer the relevant scientific questions. However, it is limited for batch analysis and (semi‐)automated treatments. In this article, we show how the framework can be used to customise the analysis to the problem at hand. We first present some possible extensions, and then we provide a simple example of automatic clustering, using the flexible Python scientific libraries which will allow to define more custom workflows in the future.

## 1 INTRODUCTION

Hydrated cementitious materials are a perfect example of a challenging material to analyse by SEM‐EDS due to their inhomogeneity, at a scale equal or lower than the interaction volume. It means than more often than not, one does not detect a phase, but a mixture of phases. Therefore, a lot of the generic tools developed for phase separations have strong limitations for cementitious materials such as the inability to detect sub‐resolutions phases and correctly identify mixture of phases.^1^

**FIGURE 1:**
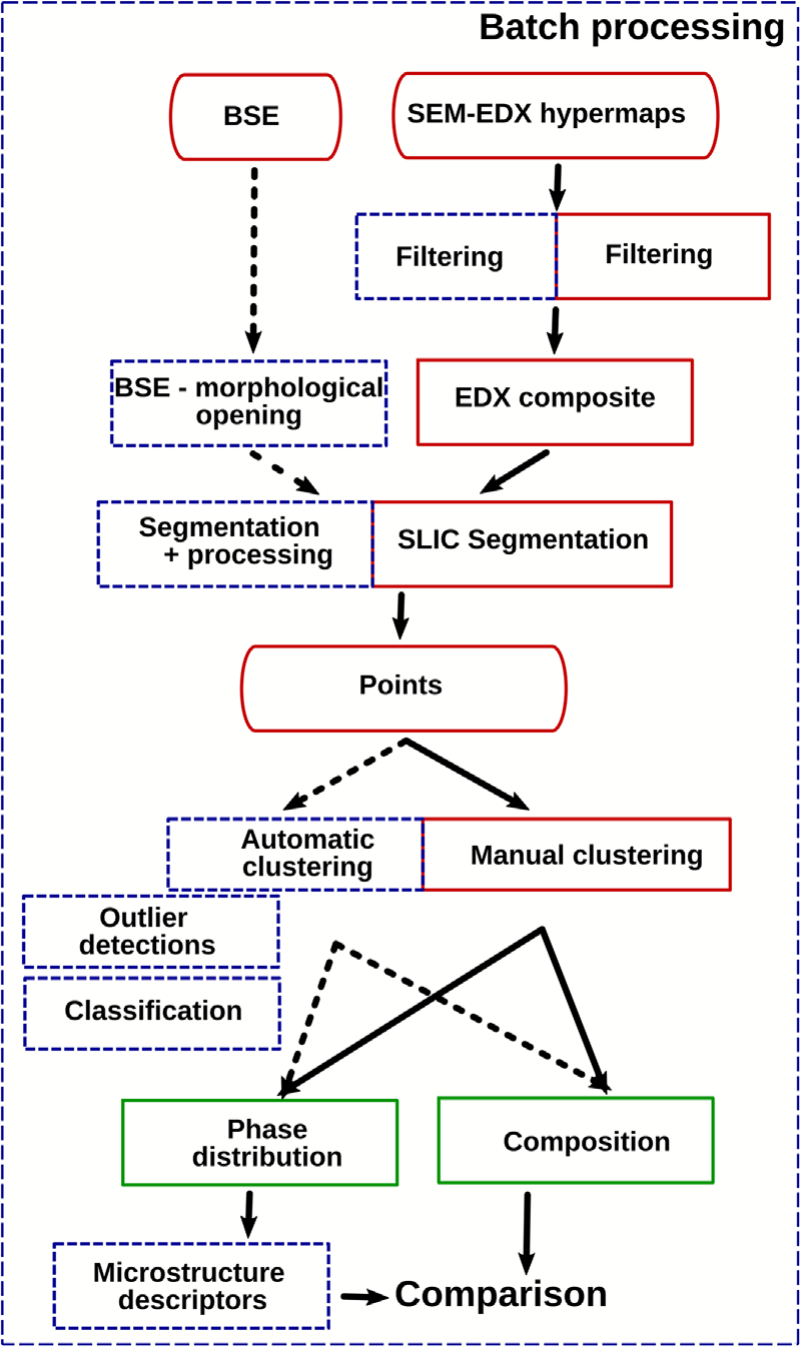
The *edxia* ^1^ flow chart. Green boxes represent the desired outputs. The solid red boxes are the original framework presented in 1. The blue dashed boxes are the extension presented in this article

To work around this issue, we developed a manual approach, which relies on the expert knowledge to perform the phase segmentation. This approach is shown in the flowchart of Figure 1, following the solid red boxes. First, a composite image is created from the main EDS maps. Then, this image is segmented using a superpixel algorithm to build a dataset of representative points. Then, these points are plotted in ratio plots, where the axes are ratio of elements such as Al/Ca versus Si/Ca. These representations are widely used in cement science, and the positions of the pure phases are easily recognised by cement experts.

The *edxia* framework is available as a plugin to the Glue software,^2^ a general purpose linked view visualisation software. This plugin is important as it give any cementitious expert without programming knowledge the ability to obtain more from their experiments. However, *edxia* is also available as an application programming interface (API), where the user can build a custom program using the edxia building blocks. The API is less accessible for a first‐time user, and thus it was not presented in the previous article to focus on the scientific aspects. This article aims to fill this gap.

## 2 MATERIALS AND METHODS

A LC^3^ (limestone calcined clay) cement paste is studied as an example. The sample preparation is detailed in the first paper.^1^, ^3^ The SEM‐EDS hypermap was acquired with a Zeiss Schottky SEM equipped with a Gemini®2 column, and an Oxford UltimMax 170 EDS detector having a silicon drift detector surface of 170 mm^2^. The hypermap was quantified using the Oxford Aztec software, using calibrated standards. Acquisition and quantification parameters are presented in detailed in the same paper.^1^ The analysis is carried out using the *edxia* framework.^1^, ^4^, ^5^

### 2.1 The scientific Python environment

The *edxia* framework is written in the Python programming language. Although originally designed as a general programming language, Python has gained a lot of attraction in the scientific community. Its user‐friendly syntax and general ease‐of‐use compensate the slower execution time compared to compiled languages such as C++. In addition to the core language, the abilities of Python are augmented by libraries or modules of ready‐to‐use functions.

For scientific abilities, most of the available libraries are based on Numpy,^6^ a library that adds support for large and multi‐dimensional arrays and matrices, and common operations on these objects. In addition, the compatible Scipy^7^ library provides the advanced numerical algorithms, such as those proposed by the well‐known C/Fortran algorithms collected in the Netlib repository. The capabilities of the Scipy library are augmented by domain‐specific libraries. The matplotlib^8^ library is one of the main libraries for visualisation. The Glue software is built on top of the latter. Scikit‐image^9^ provides the basic image analysis method (such as IO operations, filter, segmentation etc.). For extension towards machine‐learning, the scikit‐learn^10^ library can be used. These libraries ensure that no basic algorithms need to be implemented; instead the user can use these building blocks to easily implement its own data analysis flowchart, such as the one shown in blue in Figure 1.

### 2.2 Steps towards an automated process

#### 2.2.1 Segmentation of the BSE image

**FIGURE 2:**
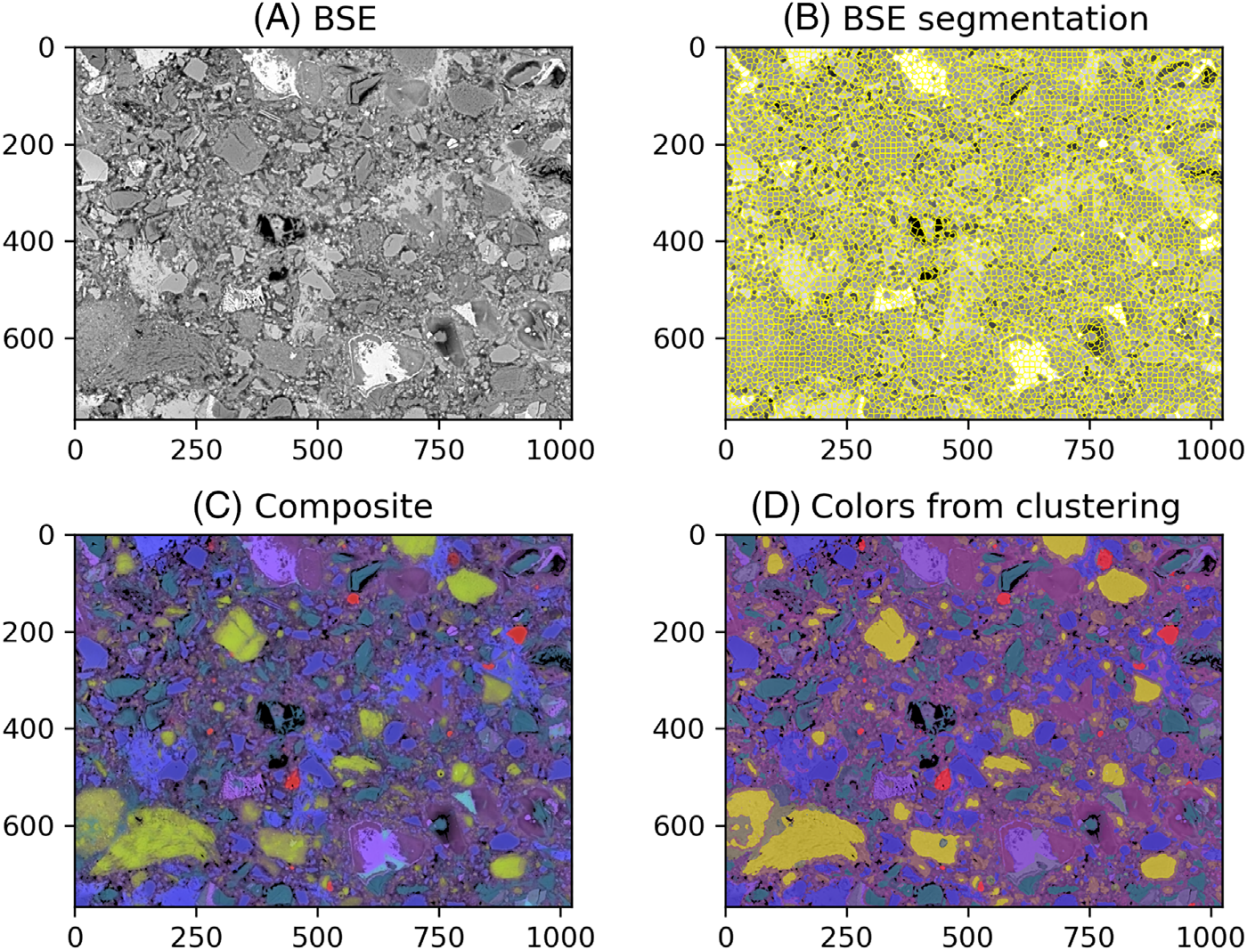
(A) BSE of the analysed sample, (B) segmentation of the BSE image, (C) composite image (Si, Ca, Al maps on top of the BSE) and (D) fake colouring of the BSE from the clustering

A critical step of the edxia algorithm is to obtain representative points of the microstructure.^1^ In the original algorithm, these points were obtained by a segmentation of a composite image made from the three main elemental EDX maps (Si, Al and Ca). This choice was made to be consistent with the EDX resolution. However, it is also possible to use the BSE image to match the grains boundaries and thus separates the macroscopic particles, or assemblages of particles. The BSE image is very fine and detailed compared to the EDX maps (see Figure 2). To better represent the resolution of the EDX hypermap, a morphological opening can be carried out on the BSE image. This filtered image can then be segmented similarly to the composite image. As it is issued from the BSE image, this segmentation better follows the visible grains, in particular the anhydrous grains. An example of this process is presented in the last part of the paper.

#### 2.2.2 Dimensionality reduction

EDX data are high‐dimensional data. Although ratio plots allows to plot 3 dimensions on a two dimensional plot, it is not sufficient to include all the information such as minor elements (Fe, Mg etc.) and the BSE. Dimensionality reduction methods^10^ have been developed to solve this challenge. They work by projecting the data on a new basis, where the variance of the data is explained by as few axes as possible. The most common method is the principal component analysis (PCA).

**FIGURE 3:**
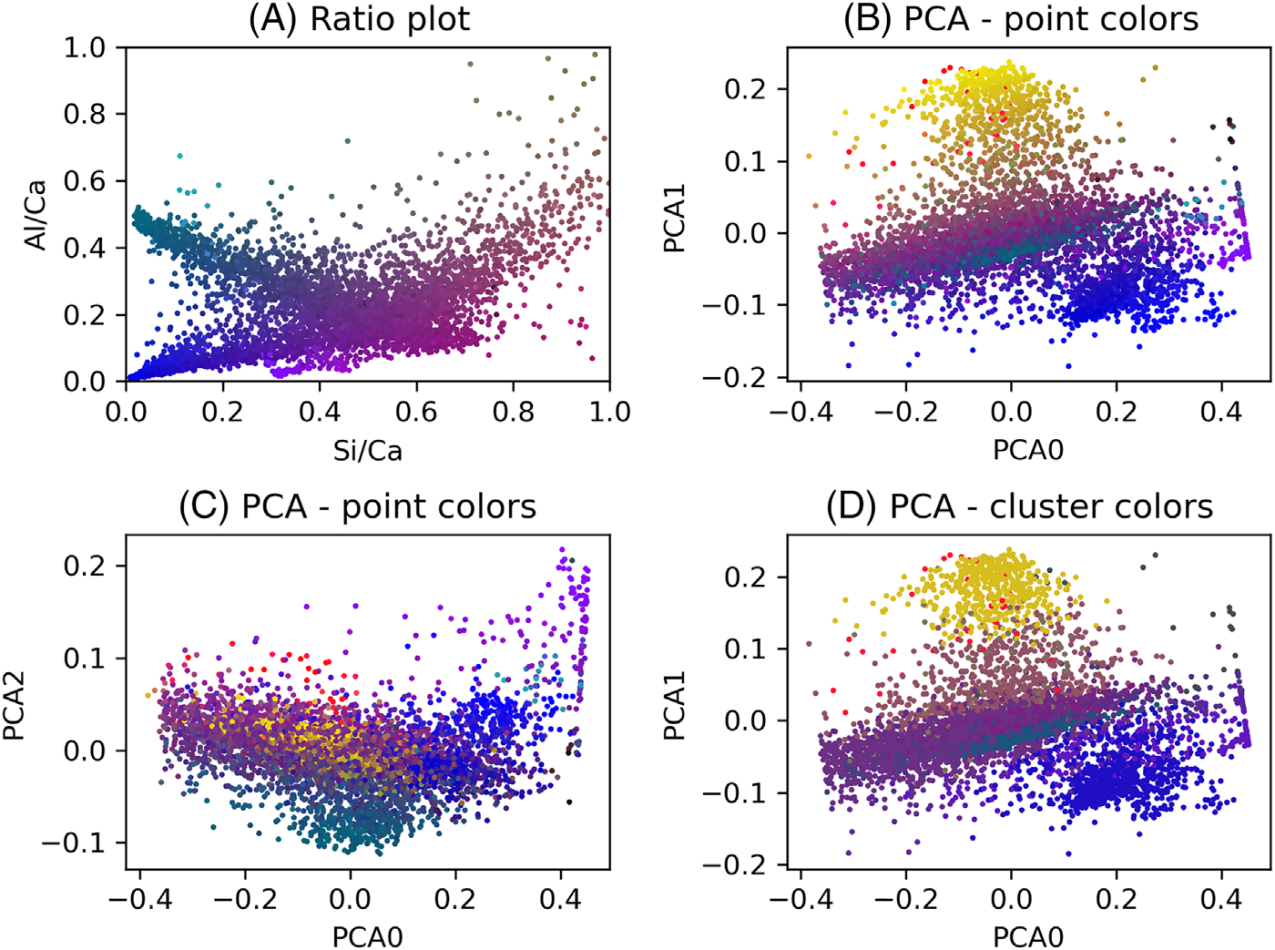
Ratio plots, (A) according the physical atomic ratios, (B)–(D) using the PCA values. For (A)–(C), colours are the colours of the composite image. For (D), colours are the median colour of the corresponding cluster

Once the representative points are obtained, the decomposition can be run. As the number of point is limited, it is a fast process. However, this transformation can then be applied to the full dataset. An example of PCA decomposition is presented in the next section and Figure 3. Unlike the ratio plots, the information is easier to visualise. For example, in the traditional ratio plots, metakaolin points tend towards a very large number. However a clear downside is that it is now much harder for an experimental expert to quickly identify the clusters. Using Glue, it is still possible to identify the clusters through the linked‐views, but it is less intuitive.

Many dimensionality reduction algorithms exist. However, they all have the drawback of breaking the physical meaning behind the axis. In addition, if ones want to understand the contribution of a minor element, the respective composition must be scaled beforehand, which breaks furthermore the physical meaning. Our linked‐view approach using Glue allows to easily coming back to the physical meaning of the mathematical meaning.

#### 2.2.3 Automatic clustering

Clustering is one of the main types of problems in machine learning. Taking a dataset, a clustering algorithm separates the points into groups. The K‐means or the Gaussian mixture algorithms are among the most common clustering algorithms.^10^, ^11^, ^12^, ^13^ Most of these algorithms take the desired number of clusters as an input. Ones could think that using the number of stable phases as the number of clusters. However, some phases (such as ettringite) are not easily visible by EDX, and others are not pure (such as the outer C‐S‐H mix). Therefore, it might be difficult to associate a cluster to a specific phase.^1^ Once again, the linked‐view approach is a good method to check the results of the algorithm. Results are presented in Figure 2. Although it is not perfect, it is sufficient to identify the main phases visible at the micron scale.

#### 2.2.4 A simple script for automatic segmentation

In this section, we describe a simple script implementing the ideas of the previous section. The full script including visualisation and automated start of the Glue software is available.

#### 2.2.5 Loading the map



data_path = <path to files>

pattern = os.path.join(data_path, “{component} Wt_.csv”)

exp = MappingExperiment(pattern, label = “LC3 28d”,

description = “LC3”,

bse_format = imagej_ascii_bse_format,

map_format = aztec_ascii_map_format)

loader = PickleLoader(exp, filters = [DenoiseFilter(0.1),])



The first step is to create the Python objects that will contain the information on the map. There are two main objects, the exp object that contains basic information about the location and format of the data and the loader object, which allows loading the data as a numpy array, after applying a filter. In this case, this is the default filter, which corresponds to the total variation algorithm, with a parameter equal to 0.1.

### 2.3 Creating the composite image



channels = CompositeChannels([“Si”, “Al”, “Ca”], [3, 4, 2])

composite = loader.load_composite(channels)

bse = loader.load_edsmap(“BSE”)

composite.mix_with_bse(filter_bse, 0.6)

composite.map[filter_bse.map<0.2,:] = 0



The next step is to create the composite image. The first line indicates which elements are associated to which colour (red, green and blue in order) and the relative multiplication factor. Using this information, the composite is created by the loader. This composite only contains EDS information, but it is possible to also mix it with the BSE image. For this purpose, the BSE image is loaded into memory. Then the composite is mixed with the BSE (with a ratio 40/60 for BSE/EDS). Finally, the pore section (BSE intensity less than 0.2) is masked in black in the composite to remove some noise.

### 2.4 Representative points from the BSE



image = minimum(maximum(img_as_ubyte(bse.map), disk(3)), disk(3))

bse_filter = Map(“Filtered BSE”, image, exp)

segmentation_labels = SlicSegmenter(0.1, 6000).apply(bse_filter)

# segmentation_labels = SlicSegmenter(0.01, 20000).apply(composite)



This section concerns the creation of the representative points from the BSE. The first line corresponds to the morphological opening of the BSE image to create BSE easier to segment. The second line wraps this layer so that it is compatible with the edxia interface. Finally, the third line calls the SLIC segmentation algorithm that will create the representative points. The last commented line can be used to segment the composite instead. However, defining representative points on the BSE ensures that homogeneous regions are created.

### 2.5 Load EDS points and points



extras = {}

stack = loader.load_stack()

extras[“SOX”] = stack.sum_of_oxides_from_mass()

stack.to_atomic(copy = False)

pts = points_from_segmentation(stack,

segmentation_labels, mask_img = composite.map,

include_rc = False, include_yx = False)



The next step is to load the full set of EDS data. This is done by the second line. This set of data is in unit of mass. Therefore, the third line is used to compute the sum of oxides, and the fourth line transforms the data in atomic unit. Finally, the last line creates the corresponding dataset of points from the segmentation.

### 2.6 PCA decomposition



decomp = PCA(8)

pca_point_res = decomp.fit_transform(pts.df)

flat = stack.flatten()

pca_stack_res = decomp.transform(flat)



This section carries out the PCA decomposition. First, it initialises the PCA algorithm, by using decomposition along four axes. The next line computes the decomposition from the representative points. The third and last lines apply this transformation to the full images, after transforming the 2D images into 1D data. This two‐step process accelerates the calculations, as the point dataset is significantly smaller than the images dataset.

### 2.7 Gaussian mixture



nc = 15 # number of components

model = GaussianMixture(n_components = nc)

gm_point_labels = model.fit_predict(pca_point_res)

gm_stack_labels = model.predict(pca_stack_res)



The last section concerns the Gaussian mixture. In this example, we use 15 clusters. The second line defines the model. The third line fits the model using the points data, and the fourth line applies this model to the EDS stack

## 3 DISCUSSION AND CONCLUSION

As presented in Figure 3, the BSE is coloured using a physically meaningful information as shown in the Si/Ca versus Al/Ca ratio plot. Using both the BSE segmentation and the EDX clustering, it is now possible to assign mean composition to macroscopic grains, and using the EDXIA tooling to assign a phase or a mixture of phase to these grains. This information can be used as a guide for the analysis presented previously,^1^ or it can help developing new workflows, for example to analyse the distribution of grains in the cement paste microstructure or to analyse patterns in the grain composition. These analyses can be reinforced using information from other sources (e.g. Raman, XRD, nano‐indentation etc.) due to edxia's generic and open Python framework
